# CDFA: Calibrated deep feature aggregation for screening synergistic drug combinations

**DOI:** 10.3389/fphar.2025.1608832

**Published:** 2025-07-23

**Authors:** Xiaorui Kang, Xiaoyan Liu, Quan Zou, Tiantian Li, Ximei Luo

**Affiliations:** ^1^ Faculty of Applied Sciences, Macao Polytechnic University, Macau, China; ^2^ Faculty of Computing, Harbin Institute of Technology, Harbin, Heilongjiang, China; ^3^ Institute of Fundamental and Frontier Sciences, University of Electronic Science and Technology of China, Chengdu, Sichuan, China; ^4^ Editorial Office, Geriatric Hospital of Nanjing Medical University, Nanjing, Jiangsu, China; ^5^ Yangtze Delta Region lnstitute (Quzhou), University of Electronic Science and Technology of China, Quzhou, Zhejiang, China

**Keywords:** drug combination, deep learning, feature fusion, transformer, synergistic drug

## Abstract

**Introduction:**

Drug combination therapy represents a promising strategy for addressing complex diseases, offering the potential for improved efficacy while mitigating safety concerns. However, conventional wet-lab experimentation for identifying optimal drug combinations is resource-intensive due to the vast combinatorial search space. To address this challenge, computational methods leveraging machine learning and deep learning have emerged to effectively navigate this space.

**Methods:**

In this study, we introduce a Calibrated Deep Feature Aggregation (CDFA) framework for screening synergistic drug combinations. Concretely, CDFA utilizes a novel cell line representation based on the protein information and gene expression capturing complementary biological determinants of drug response. Besides, a novel feature aggregation network is proposed based on the Transformer to model the intricate interactions between drug pairs and cell lines through multi-head attention mechanisms, enabling discovery of non-linear synergy patterns. Furthermore, a method is introduced to quantify and calibrate the uncertainties associated with CDFA’s predictions, enhancing the reliability of the identified synergistic drug combinations.

**Results:**

Experiments results have demonstrated that CDFA outperforms existing state-of-the-art deep learning models.

**Discussion:**

The superior performance of CDFA stems from its biologically informed cell line representation, its ability to capture complex non-linear drug-cell interactions via attention mechanisms, and its enhanced reliability through uncertainty calibration. This framework provides a robust computational tool for efficient and reliable drug combination screening.

## 1 Introduction

Drug combination therapy has emerged as a mainstay in the clinical treatment of various cancers (Meng et al., 2023), including lung cancer (Nair et al., 2023; Cui et al., 2024), ovarian cancer (Kong et al., 2023), and pancreatic cancer (Jaaks et al., 2022). Compared with monotherapy, combination therapies often demonstrate enhanced efficacy, reduced drug resistance, and decreased toxicity. However, it is crucial to recognize that not all drug combinations yield synergistic effects; in fact, some combinations may even exhibit antagonistic effects (Wang T. et al., 2023). For instance, the concomitant administration of antibiotics inhibiting DNA synthesis and those targeting protein synthesis can stimulate bacterial growth (Bollenbach et al., 2009). Therefore, the precise identification of synergistic drug pairs for specific cell types is essential to harness the full potential of combination therapy (Wang T. et al., 2022).

Traditional laboratory experiments to screen for synergistic drug combinations from the vast pharmacological space are often time-consuming and resource-intensive. Moreover, drug combination trials can sometimes result in side effects or harmful reactions in patients. With the growing availability of high-throughput screening data (Jiang et al., 2024; Liu et al., 2021), computational methods have emerged as efficient preclinical strategies for identifying synergistic drug combinations (Cao et al., 2024).

With the accumulation of data and the advancement of related technologies in recent decades, classical machine learning (ML)-based approaches and deep learning (DL) techniques have been employed to model drug combination trials, showing promising results by leveraging a variety of drug and cell line features. As drug combination effect prediction can be formulated as a regression or a multi-class classification task, the early ML-based methods often used the classical machine learning, such as logistic regression (LR) (H et al., 2014), support vector machine (SVM), random forests (RF) (Breiman, 2001), and extreme gradient boosting (XGboost). As early as 2014, Huang H et al. used a logistic regression model to systematically predict the drug combinations based on clinical side-effect (H et al., 2014). Pavel Sidorov et al. predicted Synergism of Cancer Drug Combinations by using NCI-ALMANAC Data based on RF and XGboost models (Sidorov et al., 2019). These methods laid the groundwork for more advanced approaches. Recently, deep learning (DL) models have shown excellent performance in bio-sequence analysis, gene regulation, and other areas, for extracting various data features and fusing heterogeneous data (Wang T. et al., 2024; Zhu et al., 2025). As the data about drugs continues to expand, most ML-based work has shifted towards deep learning (DL) models, driven by significant advancements in neural network architectures. One notable early DL model is DeepSynergy (Preuer et al., 2018), which integrates genomic data and drug information to identify drug combinations by a fully connected neural networks. Building on this foundation, newer DL models have emerged, leveraging advanced architectures like Transformers (Wang T. et al., 2024), Graph Neural Networks (GNNs) (Zhang et al., 2024), and Auto-Encoders (Zhu et al., 2025). For instance, CCSynergy (Hosseini and Zhou, 2023), GTextSy (Yan and Zheng, 2024), MMGCSyn (Zhang et al., 2025) and MatchMaker (Kuru et al., 2022) are integrated DNN with drug and cell line features. Based on Transformers models, DeepTraSynergy (Rafiei et al., 2023) and TranSynergy (Liu and Xie, 2021) were developed to learn drug representations and incorporate auxiliary knowledge through a novel neural network design. MRHGNN (Chen et al., 2025) and DeepDDS (Wang JX. et al., 2022) employ various GNNs to extract drug features by modeling drugs as graphs, capturing their structural properties. Moreover, recent research has introduced hypergraph neural networks to model complex relationships between cell lines and drug pairs (Wang W. et al., 2024; Liu et al., 2022).

In addition to neural network design, the fusion mechanism plays a crucial role in drug combination synergy prediction models. Recent studies have focused on effectively combining drug and cell line information to improve predictive accuracy. In parallel, advances in biological sequence classification have demonstrated the benefits of integrating multiple types of information. For instance, the SBSM-Pro model (Wang YZ. et al., 2024) introduces a novel multiple kernel learning strategy to combine sequence similarity measures, significantly enhancing classification performance. Similarly, DFFNDDS (Xu et al., 2023) employs two distinct neural networks to fuse drug features and cell line information from both bit-wise and vector-wise perspectives. DualSyn (Chen et al., 2024) introduces two modules to capture high-order and global information, enhancing the model’s ability to understand complex interactions. SynergyX (Guo et al., 2024) utilizes mutual-attention and self-attention mechanisms to model drug-cell and drug-drug interactions, providing a more nuanced understanding of these relationships. CircRDRP (Wang Y. et al., 2024) uses a graph neural network model to predict the association of circRNA with drug resistance by combining disease context characteristics and deep learning techniques. MMSyn (Pang et al., 2024) and AttenSyn (Wang TS. et al., 2023) leverage attention mechanisms to integrate multiple drug and cell line features, allowing the model to focus on the most relevant aspects of the data. CLCDA (Wang YT. et al., 2023) is a collaborative deep learning-based model for predicting potential associations between circRNA and disease. Despite these significant contributions, many of these approaches still rely on late fusion mechanisms, where drug and cell line features are combined at a later stage in the model. This can limit the model’s ability to fully capture the intricate interactions between drugs and cell lines. To address the limitations of late fusion mechanisms, this study proposes the Calibrated Deep Feature Aggregation (CDFA) framework–a Transformer-based architecture that enables early-stage integration of proteomic features and gene expression profiles to capture intricate drug-drug-cell interactions. The design incorporates dedicated uncertainty calibration to ensure probabilistic reliability. Experimental validation demonstrates CDFA’s fusion efficacy: comprehensive testing across two benchmark datasets (spanning diverse cell lines and tissue types) confirms both the structural effectiveness and superior generalization of our approach.

## 2 Materials and methods

### 2.1 Synergy datasets

**FIGURE 1:**
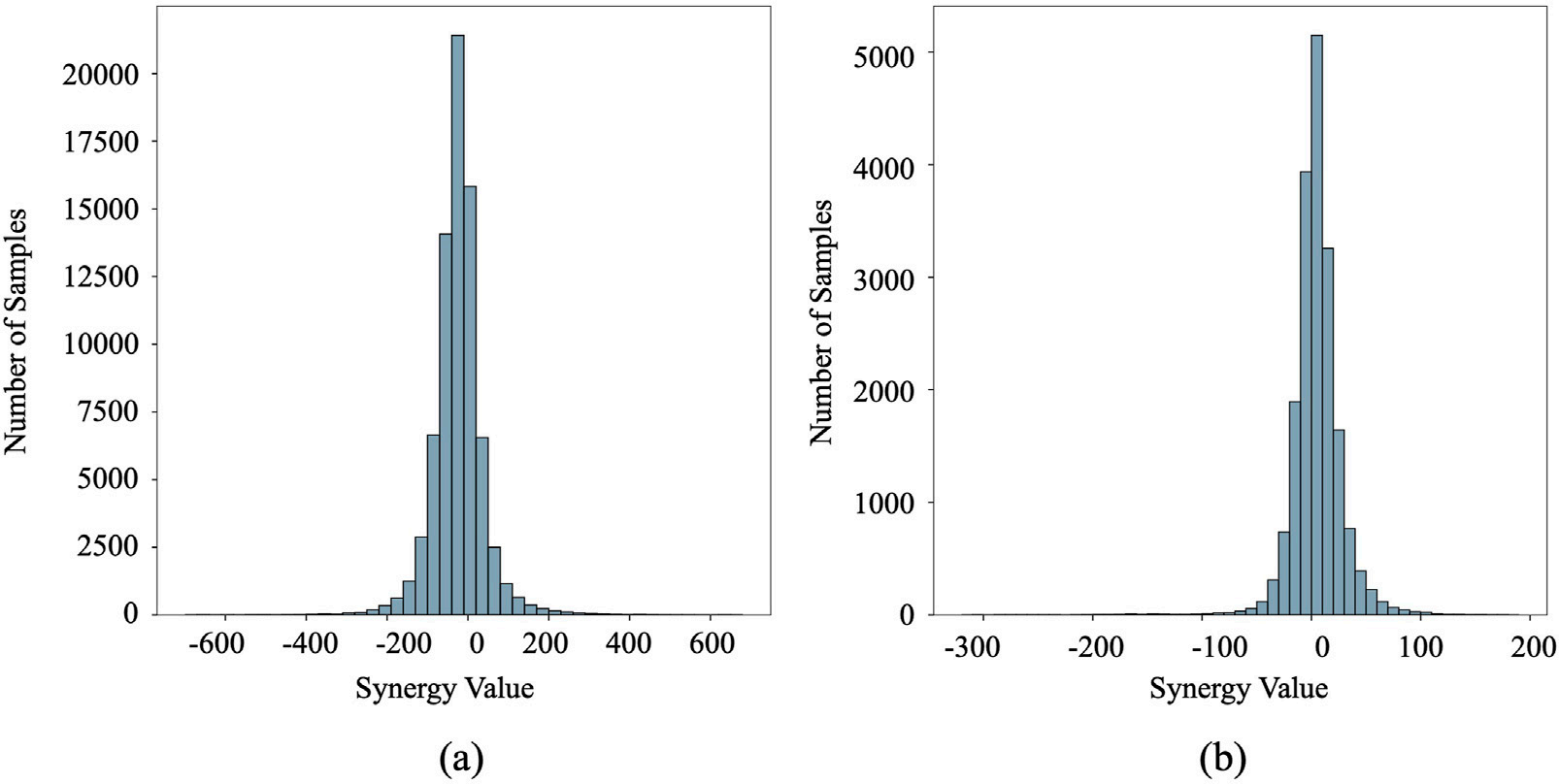
The distribution of synergy scores in the O’Neil and NCI-ALMANAC datasets. The vertical axis represents sample frequency counts, while the horizontal axis displays synergy scores. **(a)** O’Neil dataset. **(b)** NCI-ALMANAC dataset.

We assessed our method using two publicly available datasets: O'Neil (O'Neil et al., 2016) and NCI-ALMANAC (Holbeck et al., 2017). The O'Neil dataset comprised 23,062 drug combination samples involving 38 drugs and 39 human cancer cell lines. The NCI-ALMANAC dataset was relatively larger, containing 304,549 data points across 104 drugs and 60 cell lines. The synergy value for each sample is represented by the Loewe and combination scores for O'Neil and NCI-ALMANAC, respectively. The characteristics of the cell lines were represented by 651 gene expression values obtained from the COSMIC database (Forbes et al., 2015). Following established preprocessing steps (Liu et al., 2022), the final datasets included 18,950 and 74,139 drug-drug-cell line combinations for O'Neil and NCI-ALMANAC, respectively. Figure 1 depicts the distribution of synergy scores for both datasets. Notably, the left side of the distribution, centered around 30, constitutes more than half of the dataset. These values correspond to the negative pairs that exhibit either additive or antagonistic effects, indicating that a significant portion of the drug combinations do not show a synergistic benefit over the individual effects of the drugs. This observation underscores the complexity of identifying truly synergistic drug pairs and highlights the importance of systematic screening and computational approaches to optimize drug combination therapies.

To train and evaluate the model, we began by randomly selecting 90% of drug pairs and cell lines from each dataset to conduct three different experimental settings: random setting, cold cell line setting, and cold drug pair setting. The remaining 10% of the samples were set aside as an independent test set to evaluate generalization performance. For the random splitting setting, we divided the samples into five equal subsets. One subset served as the test set, while the remaining four were further split into training and validation sets in a 9:1 ratio. In the cold cell line setting, all the unique cell lines were divided into five equal groups randomly. The related samples which contain the cell line from one of these groups were used for testing, while the remaining samples were split into a 9:1 ratio as the training set and validation set. This ensured that the test set included only cell lines not present in the training set. For the cold drug pair setting, drug pairs were similarly partitioned into five equal groups. Four groups were used for training, with the test set containing only those drug pairs not seen during training. This ensured that the model was tested on an entirely new pair of drugs.

### 2.2 Problem formulation

In this study, we formulate the synergy prediction problem as a regression task. Let $X_{\mathrm{train}}=\{d_{1}^{i},d_{2}^{i},c^{i}{\}}_{i‐1}^{N}$ denote the set of the training samples where $d_{1}^{i},d_{2}^{i}$ denote the drug pair and $c^{i}$ is the cell lines, and N denotes the number of training samples. Also, the corresponding synergy effect is represented by the label $Y_{train}=\{y^{i}{\}}_{i=1}^{N}$ . The paper aims at learning a drug combination function $f\left(\cdot \right)$ , given a drug pair and a cell line, $f\left(\cdot \right)$ can generate the target value $\hat{y}$ .

### 2.3 Drug and cell line representations

A variety of molecular representations have been employed for drug combination prediction tasks. Fingerprints, such as ECFP and MHFP, are commonly used to encode compound structures. In this study, we adopted the MinHashed Atom-Pair fingerprint extended to four bonds (MAP4) as our molecular representation. MAP4 offers a versatile approach to representing diverse chemical structures.

Gene expression profiles have been commonly employed to represent cell lines in drug combination prediction tasks. In this study, we utilized gene expression data extracted from COSMIC, represented as 651-dimensional vectors ( g ), where each element corresponds to the expression level of a specific gene. In the most of the deep learning-based models treat the gene expression g as a vector which does not satisfy the biomedical meaning which each gene expression should be treated separately. In the bio-mechanism of drug synergy, only a part of genes contributes to the synergy effect. So, we treat the 651-dimensional vectors ( g ) as a matrix $H=\{h_{j}{\}}_{j=1}^{651}\in R^{1\times 651}$ . In the following work, we use the CNN to extract the important genes to simulate the bio-mechanism.

### 2.4 Feature encoder

The weighted gene expression representation of a cell line is fed into a cell line feature encoder to learn abstract cell line representations. This encoder comprises three convolutional layers interleaved with pooling layers. The initial convolutional layer transforms the input into feature maps, which are subsequently downsampled using max-pooling. This process is repeated three times.

The MAP4 vector representing a drug is input into a drug feature encoder to extract high-level abstract features. The encoder consists of two fully connected (FC) layers followed by Gaussian Error Linear Units (GELU) (Hendrycks and Gimpel, 2016) and batch normalization. The resulting features serve as essential inputs for subsequent fusion operations. The formulation of the drug feature encoder can be summarized as follows (Equation 1):

$$F_{o}^{i}=BN\left(GELU\left({FC}^{1024}\left(BN\left(GELU\left({FC}^{2048}\left(x_{o}^{i}\right)\right)\right)\right)\right)\right)$$ (1)

where $x_{o}^{i}$ is one of the input features and $F_{o}^{i}$ denotes the corresponding generated feature. BN represents 1day batch normalization. ${FC}^{n}\left(\cdot \right)$ represents an FC layer with n neurons. During the feature extraction stage, we project drug features and cell line feature into the same dimension to obtain higher-quality information for use in the subsequent modules.

We refer to these generated drug pair features as $\left(F_{d_{1}},F_{d_{2}}\right)\in R^{D}$ and cell line feature as $F_{c}\in R^{L\times D}$ .

### 2.5 Deep feature aggregation module

Given the drug pair features $\left(F_{d_{1}},F_{d_{2}}\right)\in R^{D}$ and extracted feature of weight gene expression of cell line $F_{c}\in R^{L\times D}$ , we first use a global max pooling operation to obtain the global cell line feature as $G_{c}\in R^{D}$ . We treat the drug pair features and global cell line feature as whole global features $G=\left\{F_{d_{1}},F_{d_{2}},G_{c}\right\}\in R^{3\times D}$ and the $F_{c}$ as the local cell line feature. The deep feature aggregation module can be decomposed into two parts: 1) global feature fusion, and 2) global to local feature fusion. Details are discussed as follows:

*Global feature fusion*: This process aims to integrate drug and early cell line features, followed by reinforcing the fused global features back into the local cell features. We employ a transformer encoder for global feature fusion. The core idea of the transformer encoder is the attention mechanism. An attention function maps queries ( Q ), keys ( K ), and values *(* V ) to an output o as follows (Equation 2):

$$Attention\left(Q,K,V\right)=softmax\left(\frac{QK^{T}}{\sqrt{d}}\right)V$$ (2)

where $\sqrt{d}$ is the dimensionality of the query vector.

The multi-head attention mechanism consists of multiple attention heads, with each head conducting a linear transformation on the input vectors before performing the attention operation. Each attention head has its own set of trainable parameters, allowing it to potentially model an independent relationship between the input vectors. This is achieved by utilizing different parameters in the linear transformation step.

Then, for the h head, three weight matrices $W^{Qh},W^{Kh},W^{Vh}\in R_{d_{f}\times d_{p}}$ are used to project *Q*, *K*, and *V*, respectively, to a lower dimension $d_{p}$ ; then, an attention function is performed (Equation 3).

$$A^{h}=Attention\left(Q^{h},K^{h},V^{h}\right)$$ (3)

wherein $Q^{h}=QW^{Qh},K^{h}=KW^{Kh},{\;V}^{h}=VW^{Vh}$ .

Then, the output of the multi-head attention mechanism is the linear transformation of the concatenation of the output vectors acquired from the attention heads (Equation 4):

$$MultiHead\left(Q,K,V\right)=Concat\left(A^{1},A^{2},\cdots ,A^{H}\right)W^{O}$$ (4)

where H is the number of heads and $W^{O}$ is a trainable weight matrix.

Besides the attention mechanism, the transformer encoder also contains the residual and feed-forward neural network. Formally, the global feature fusion can be defined as follows (Equation 5):

$$A_{G}=\mathrm{LN}\left(G+MultiHead\left(G,G,G\right)\right)$$

$$F_{G}=\mathrm{LN}\left(A_{G}+FFN\left(A_{G}\right)\right)$$ (5)

where $\mathrm{LN}\left(\cdot \right)$ and $FFN\left(\cdot \right)$ represent layer normalization and feed-forward neural network, respectively.

*Global to local cell line feature fusion*: Inspired by recent findings that drugs can influence the synergistic or antagonistic effects of drug combinations through modulating key gene expression (Wu et al., 2023), we incorporate a global-to-local cell line feature fusion network to simulate drug-induced gene regulation effects. The local cell line feature is enhanced through multi-head attention where global features ( $F_{G}$ )are incorporated using a Transformer decoder. This enables adaptive re-weighting of gene expressions based on cross-tissue biological patterns, with layer normalization and residual connections stabilizing feature refinement. This process can be mathematically expressed as follows (Equation 6):

$$F_{G}=\mathrm{LN}\left(F_{c}+\text{MultiHead}\left(F_{c},F_{c},F_{c}\right)\right)$$

$$C_{G}=\mathrm{LN}\left(F_{c}+\text{MultiHead}\left(F_{c},F_{G},F_{G}\right)\right)$$

$$F_{c}=\mathrm{LN}\left(C_{c}+FFN\left(C_{c}\right)\right)$$ (6)

### 2.6 Synergy prediction module

The final synergy value of a drug combination is predicted using the output of the global feature fusion network ( $F_{G}$ ) and the global-to-local cell line feature fusion network ( $F_{c}$ ). Specifically, $F_{G}$ is flattened into a 1D vector, and global max pooling is applied to $F_{c}$ to obtain another 1D vector. These vectors are then fed into separate multi-layer fully connected layers to refine their abstract features. Finally, the refined features are concatenated and passed through a final FC layer to predict the synergy value $\hat{y}$ .

Given a training dataset, that contains N samples with ground-truth synergy scores y and the corresponding values $\hat{y}$ predicted by our method, we can train the deep learning model in an end-to-end fashion using the mean squared error (MSE) loss as the loss function.

### 2.7 Uncertainty quantification

We use an ensemble method to further enhance generalization and quantify the uncertainty of the CDFA. Specifically, we trained M distinct model replicas. Each replica shares the same neural network architecture and settings but uses a different initial random seed for parameter initialization. This ensures that while the models are structurally identical, they develop unique parameter values during training, leading to diverse predictions and a more robust uncertainty estimation. For every input drug combination, each model generates a predicted synergy value, denoted as ${\hat{y}}_{k}^{\left(d_{1}^{i},d_{2}^{i},c^{i}\right)}$ . The final synergy prediction, $\mu \left(d_{1}^{i},d_{2}^{i},c^{i}\right)$ , is determined by averaging these individual predictions. Meanwhile, the uncertainty associated with this prediction, $\sigma \left(d_{1}^{i},d_{2}^{i},c^{i}\right)$ , is quantified by calculating the standard deviation of the individual predictions from the ensemble.

### 2.8 Uncertainty recalibration

Calibration errors (Mervin et al., 2021) in probability estimates compromise reliability by creating discrepancies between predicted and true probabilities. Specifically, they refer to the discrepancy between the model’s predicted confidence and the actual observed frequency of correctness at that confidence level. For example, if a model assigns 80% confidence to a set of predictions, but only 70% of them are correct, this indicates a calibration error in that confidence range. Such miscalibration reduces the effectiveness of uncertainty estimates as indicators of trustworthiness in predictions.

To address this issue, a common strategy is to learn a recalibration function that adjusts the predicted uncertainties to better align with the true underlying probabilities. The recalibration function is often a non-linear uncertainty scaling function, learned using a hold-out validation dataset to create a calibration map, and is often assessed using metrics like Expected Calibration Error (ECE). In our method, we adopt a simple yet effective single-parameter scaling approach that adjusts only the uncertainty component $\sigma \left(\cdot \right)$ . We achieve this by multiplying $\sigma \left(\cdot \right)$ with a scaling factor r , while keeping the predicted synergy value $\mu \left(\cdot \right)$ unchanged. This choice is motivated by the fact that $\mu \left(\cdot \right)$ , as the model’s point estimate, already captures the optimal synergy prediction and should not be altered during post-hoc calibration. Instead, we rescale $\sigma \left(\cdot \right)$ by a positive scalar factor r , resulting in the recalibrated output $\mu \left(\cdot \right),r\sigma \left(\cdot \right)$ . The scaling factor r is optimized using Brent’s method (Brent, 1971) to ensure that the recalibrated uncertainties accurately reflect the true probability of correctness. The objective is to minimize the miscalibration, quantified by ECE, on a separate validation set. This optimization ensures that the adjusted uncertainties more accurately reflect the true likelihood of correct predictions across confidence levels. The result is an uncertainty estimate that is better aligned with the model’s empirical behavior and more trustworthy for downstream decision-making.

## 3 Results

### 3.1 Overview of the CDFA framework

**FIGURE 2:**
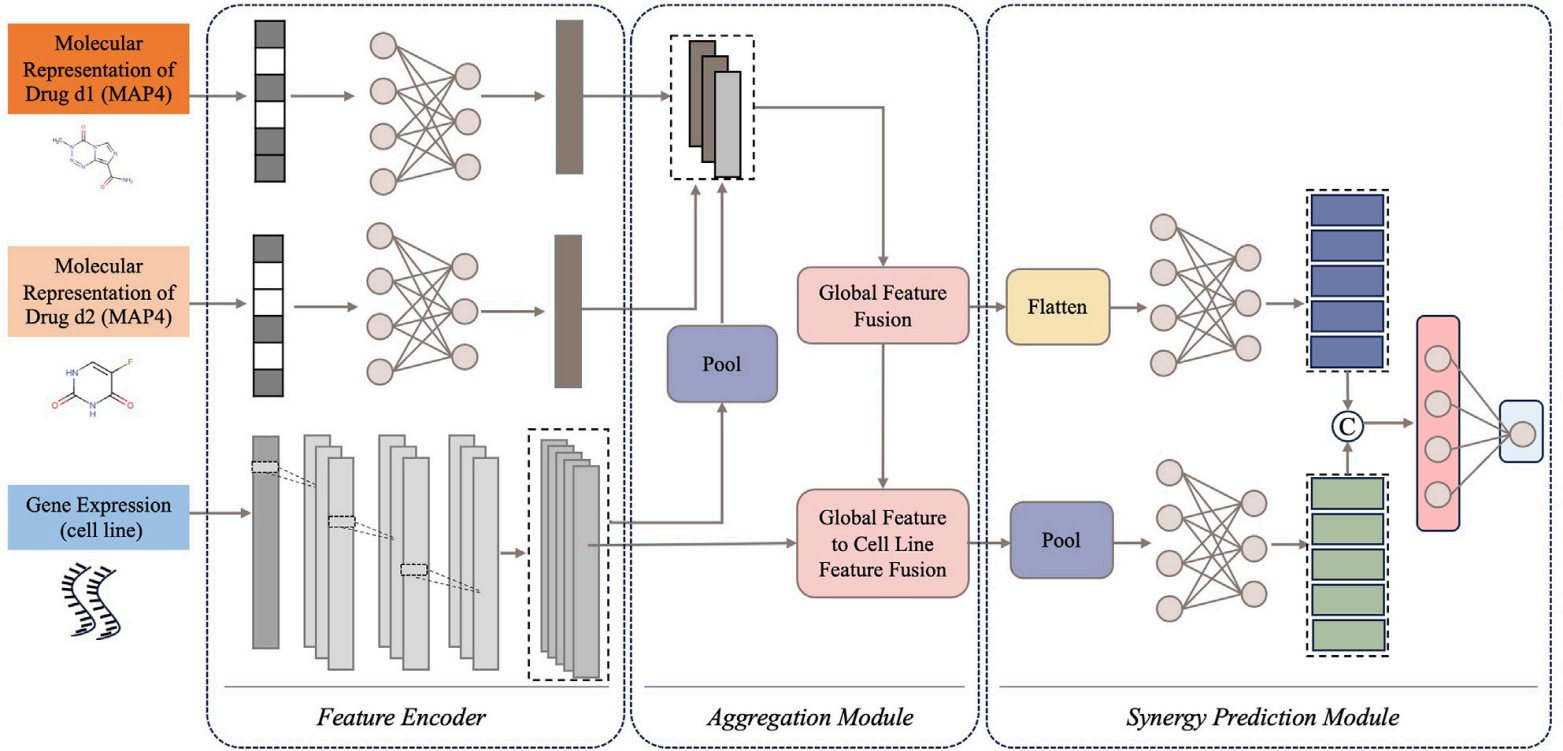
The overview network of CDFA. Data flows sequentially from input to output through three core components: (1) feature encoders for drug pairs and cell lines, (2) feature aggregation module, and (3) synergy prediction module.

CDFA is an ensemble deep learning framework for predicting the potential synergy effects of drug combinations based on the drugs’ molecular information and the cells’ gene expression. The overall architecture of CDFA is shown in Figure 2. It consists of three main components: the feature encoders for the drug pair and cell line, the feature aggregation module, and the synergy prediction module. First, MAP4 is used to represent diverse chemical structures of the paired drugs. Gene expression profiles are employed to represent cell lines in drug combination prediction tasks. Then, feature encoders are used to extract these three types of features separately. A novel feature aggregation network is involved based on the Transformer which tries to capture the intricate interactions between drug pairs and cell lines. Finally, the aggregated features are connected to another synergy prediction module. The subsequent sections of this section provide detailed evidence of the superiority of this computational framework.

### 3.2 Comparison with existing models

To evaluate CDFA’s performance, we compared it with nine existing drug combination synergy prediction models: HypergraphSynergy, DeepSynergy, DTF, CombFM, Celebi’s method, PermuteDDS, MatchMaker, GTextSyn and MMGCSyn. We employed three common regression evaluation metrics to assess the performance of these methods: root mean squared error (RMSE), coefficient of determination ( $R^{2}$ ), and Pearson’s Correlation Coefficient (PCC).

As Table 1 shows, we compared CDFA’s performance with several models using the O'Neil dataset across three different experimental setups. In the random split scenario, where data is divided without specific constraints, the CDFA model outshone others with the lowest RMSE at 13.522, alongside the highest $R^{2}$ at 0.651 and PCC at 0.808. When tested on unseen cell lines (cold cell line setting), HypergraphSynergy led with the highest $R^{2}$ of 0.252 and the lowest RMSE of 19.537. However, CDFA maintained a competitive edge despite not leading in every metric. For the cold drug pair setting, where models predict outcomes for drug combinations not encountered during training, CDFA performed exceptionally well, achieving the lowest RMSE (15.976), highest $R^{2}$ (0.511), and a PCC of 0.717, demonstrating its strength in handling unseen drug pairs.

**TABLE 1:** Performance comparison on the O’Neil dataset. Bold values indicate the best performance.

	Randon split			Cold cell line setting			Cold drug pair setting		
	RMSE	R2	PCC	RMSE	R2	PCC	RMSE	R2	PCC
CDFA	**13.522**	**0.651**	**0.808**	19.597	0.25	0.53	**15.976**	**0.511**	**0.717**
PermuteDDS	13.721	0.641	0.801	19.668	0.243	0.522	16.152	0.501	0.709
HypergraphSynergy	14.727	0.586	0.775	**19.537**	**0.252**	0.533	17.346	0.42	0.656
DeepSynergy	14.87	0.584	0.765	23.89	0.195	0.426	17.28	0.433	0.663
ComboFM	16.86	0.451	0.702	20.82	0.142	0.396	18.62	0.376	0.635
DTF	14.73	0.594	0.775	21.11	0.132	**0.535**	17.37	0.429	0.671
Celebi’s method	16.34	0.5	0.708	20.6	0.179	0.473	19.1	0.309	0.572
MatchMaker	17.4948	0.4162	0.6466	28.5376	−0.7616	0.3628	17.7172	0.399	0.6332
GTextSyn	16.231	0.497	0.709	20.866	0.144	0.457	18.186	0.367	0.625
MMGCSyn	17.138	0.439	0.69	25.754	−0.342	0.316	18.837	0.317	0.605

As shown in Table 2, the consistent superiority of CDFA has also been demonstrated on the NCI-ALMANAC dataset. In the random split setup, CDFA exhibited the best performance with the lowest RMSE of 41.893, highest $R^{2}$ of 0.552, and highest PCC of 0.746. Under the cold cell line condition, HypergraphSynergy performed best with an RMSE of 53.398, $R^{2}$ of 0.273, and PCC of 0.538. In the cold drug pair scenario, CDFA once again stood out, achieving the lowest RMSE (50.522), sub-optimal $R^{2}$ (0.346), and highest PCC (0.593), underscoring its effectiveness in predicting responses for novel drug combinations.

**TABLE 2:** Performance comparison on the NCI-ALMANAC dataset. Bold values indicate the best performance.

	Randon split			Cold cell line setting			Cold drug pair setting		
	RMSE	R2	PCC	RMSE	R2	PCC	RMSE	R2	PCC
CDFA	**41.893**	**0.552**	**0.746**	53.819	0.259	0.536	**50.522**	0.346	**0.593**
PermuteDDS	43.053	0.527	0.726	54.128	0.242	0.519	51.58	0.318	0.569
HypergraphSynergy	43.89	0.508	0.719	**53.398**	0.273	**0.538**	52.609	0.291	0.543
DeepSynergy	44.44	0.491	0.701	54.56	0.23	0.322	53.5	0.262	0.526
ComboFM	48.27	0.399	0.651	54.67	0.245	0.531	53.89	0.267	0.526
DTF	47.03	0.43	0.678	54.73	0.223	0.517	53.47	0.263	0.531
Celebi’s method	47.31	0.423	0.653	53.49	0.259	0.516	55.83	0.196	0.456
MatchMaker	51.7316	0.3168	0.5642	64.6824	**0.3644**	−0.0652	55.7034	0.2028	0.4588
GTextSyn	47.425	0.426	0.657	56.369	0.187	0.479	55.511	0.208	0.483
MMGCSyn	47.793	0.417	0.659	60.353	0.067	0.454	54.523	**0.519**	0.236

The 10% of the samples of the O'Neil and NCI-ALMANAC datasets were set aside as an independent test set to evaluate these models’ generalization performance. In the independent test data section of the O'Neil and NCI-ALMANAC datasets, the superior performance of CDFA has been once again proven. As Table 3 shows, it illustrates the performance of various methods when applied to the independent test datasets. For the O'Neil dataset, CDFA demonstrates superior accuracy with the lowest RMSE of 15.111 and the highest $R^{2}$ of 0.660. PermuteDDS trails closely behind with similarly strong results, showing almost no difference from CDFA. On the NCI-ALMANAC dataset, CDFA retains its leadership by achieving the best RMSE at 42.307 and the highest $R^{2}$ value at 0.508, confirming its robustness in both precision and explanatory capability. Although PermuteDDS performs well, it still lags slightly behind CDFA across all metrics. The remaining methods exhibit higher RMSE figures and lower $R^{2}$ values, suggesting they are less precise and less effective compared to our method.

**TABLE 3:** Performance comparison on the independent test datasets. Bold values indicate the best performance.

	Randon split			Cold cell line setting		
	RMSE	R2	PCC	RMSE	R2	PCC
CDFA	**15.111**	**0.660**	0.818	**42.307**	**0.508**	**0.713**
PermuteDDS	15.144	0.659	**0.821**	43.338	0.484	0.696
HypergraphSynergy	16.710	0.585	0.788	43.730	0.474	0.693
DeepSynergy	16.840	0.578	0.765	45.325	0.435	0.670
ComboFM	16.080	0.541	0.754	46.370	0.457	0.685
DTF	16.150	0.548	0.752	49.860	0.372	0.700
Celebi’s method	16.500	0.529	0.728	45.860	0.469	0.688
MatchMaker	20.725	0.361	0.6466	51.259	0.2778	0.5282
GTextSyn	18.931	0.466	0.686	48.026	0.366	0.612
MMGCSyn	19.834	0.412	0.647	48.312	0.358	0.619

Overall, CDFA consistently demonstrated strong performance, particularly excelling in the random split and cold drug pair settings. However, the poor performance of all methods in the cold cell line setting suggests that future research should focus on improving models' ability to generalize to new cell lines.

### 3.3 Tissue-specific analysis

Both previous studies and our own experiments have consistently demonstrated that model performance deteriorates significantly under the cold cell-line scenario, where test cell lines are entirely disjoint from those seen during training. This setting introduces substantial biological variability, making it difficult to disentangle whether performance degradation arises from tissue-specific effects or from the challenge of generalizing to unseen cell-line profiles.

**FIGURE 3:**
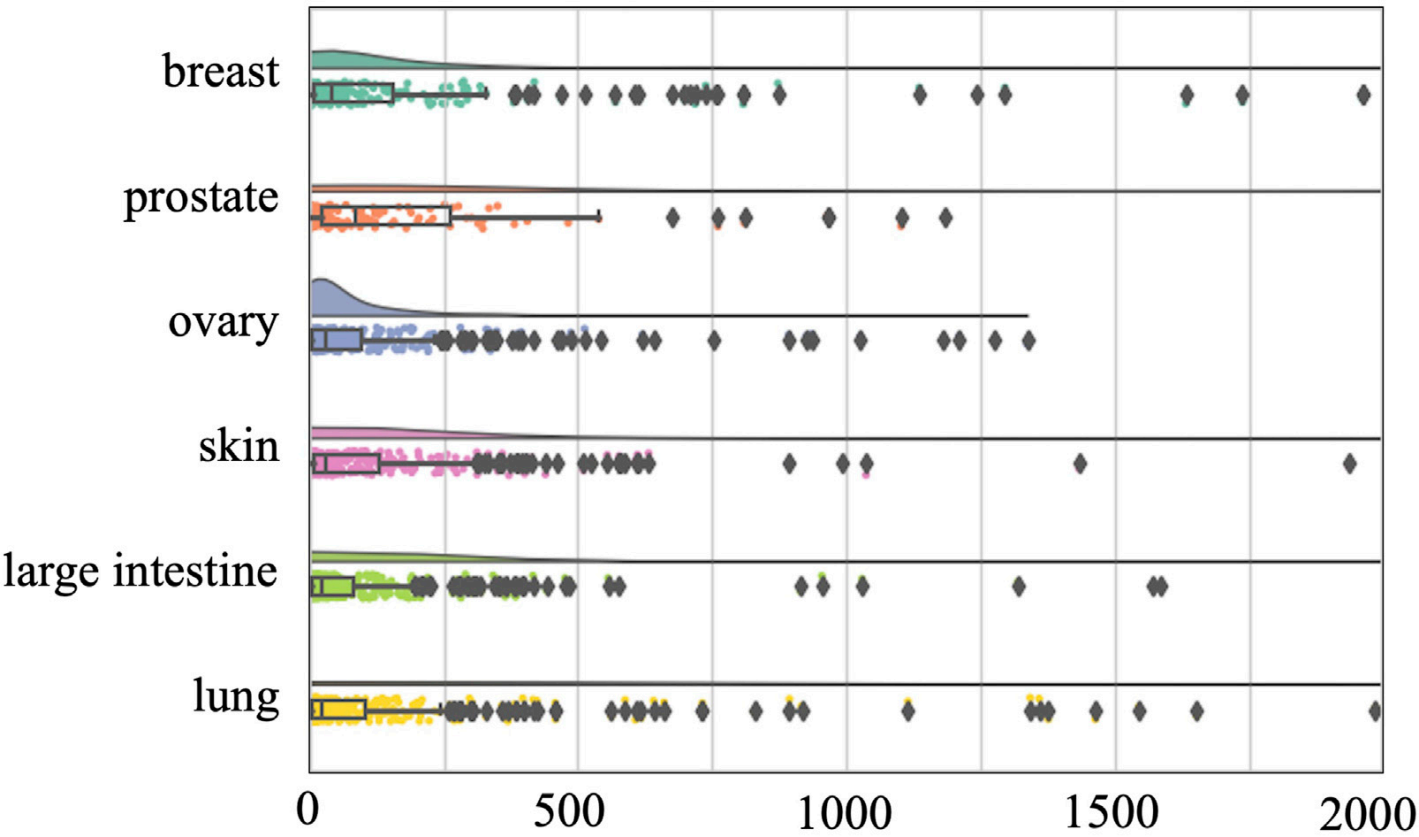
Raincloud plots of MSE for O’Neil independent test dataset. The horizontal axis quantifies mean squared error (MSE) between true synergy scores and model predictions.

**FIGURE 4:**
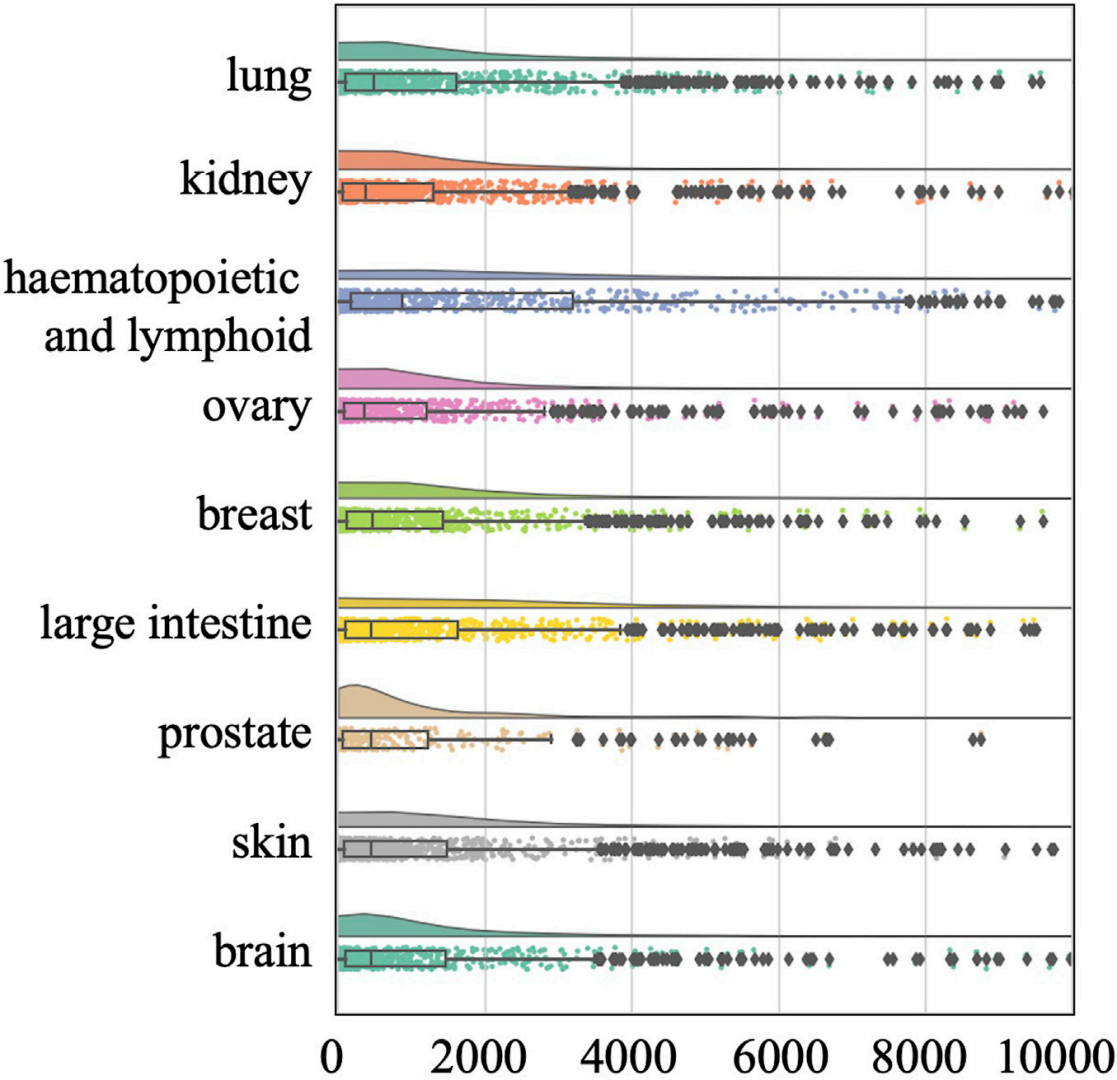
Raincloud plots of MSE for NCI-ALMANAC independent test dataset. The horizontal axis quantifies mean squared error (MSE) between true synergy scores and model predictions.

To avoid this confounding factor, we also conducted a tissue-specific analysis on the O’Neil and NCI-ALMANAC datasets. The O’Neil dataset is built on testing 38 drugs on 39 cell lines representing multiple cancer types from six tissue origins. The NCI-ALMANAC dataset covers 104 drugs in 60 cell lines from nine tissue origins. As illustrated in Figures 3, 4, our analysis employs raincloud plots to visualize the distribution of MSE for the two independent test datasets. These plots combine box plots with kernel density estimates ('clouds') to visualize both the shape and central tendency of the error distributions, with outliers indicated by diamond markers.

Our analysis reveals that although the MSE values of the median, second quartile, and third quartile are low, almost all tissues included by the two datasets have MSE values exceeding 500 and 2000, respectively. This suggests that while there is a small number of higher error values across most tissues, the central tendency of the error distribution may be relatively low. This pattern indicates that the model can achieve efficient prediction across different tissues. Our analysis confirms that the presence of high-error predictions—though limited in quantity—reveals significant variability in model performance. Such findings highlight the need for further investigation into the factors contributing to these higher errors and suggest that improvements in model accuracy and consistency are necessary for more reliable predictions across different tissue types.

### 3.4 Uncertainty results

**FIGURE 5:**
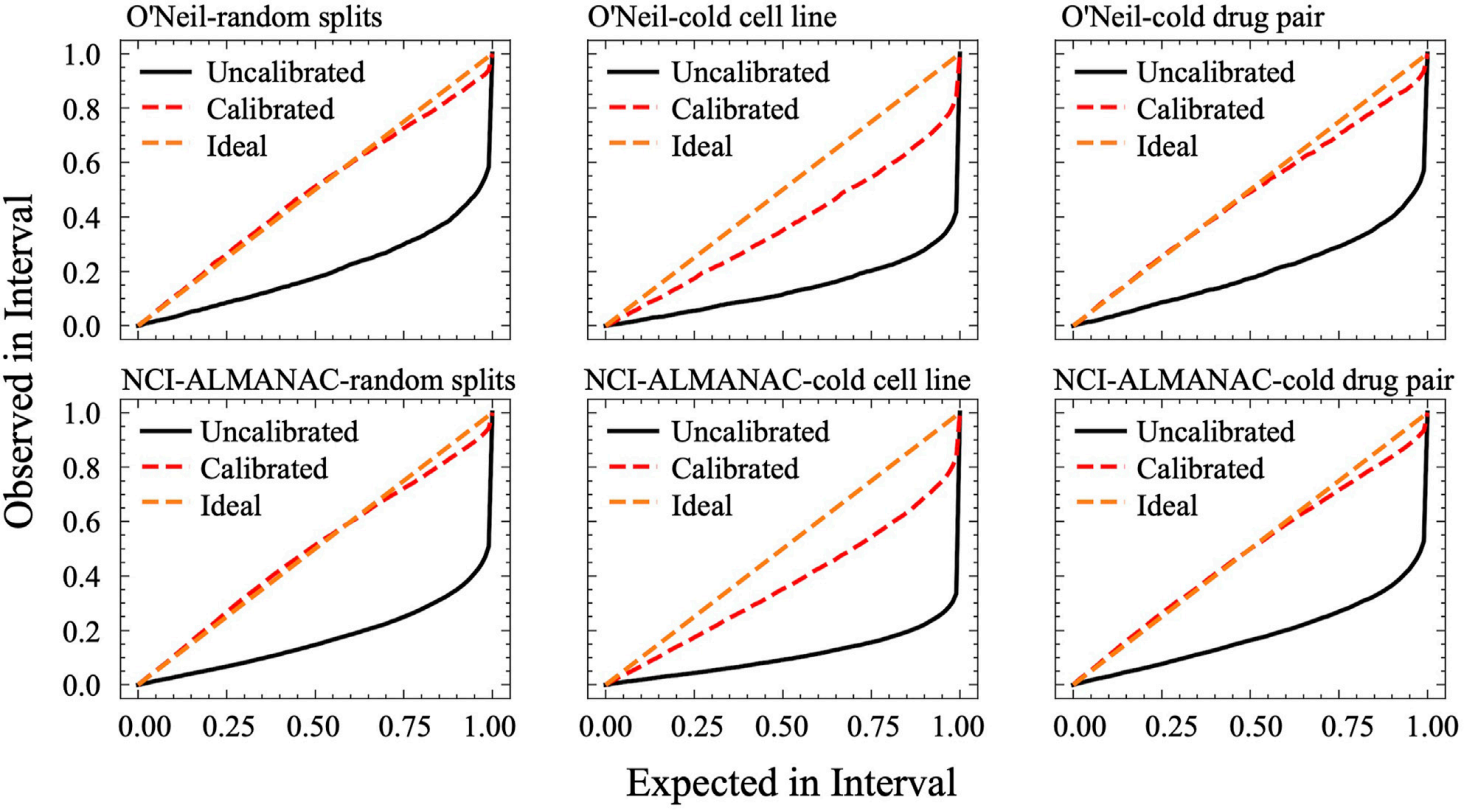
Calibration curves of CDFA. On the x-axis, it plots the expected confidence level, while the y-axis shows the observed proportion of correct predictions. A perfectly calibrated model lies on the diagonal line, where the observed proportion of correct outcomes exactly matches the stated confidence at every interval.

Figure 5 displays the calibration curves of CDFA under various settings for both the O'Neil and NCI-ALMANAC datasets. The figures are organized from left to right, representing random splits, cold cell line settings, and cold drug pair settings, respectively. The first row showcases the O'Neil dataset, whereas the second row pertains to the NCI-ALMANAC dataset. The space between the calibration curves and the diagonal line represents the miscalibration area, which quantifies the extent of uncertainty calibration. As illustrated in Figure 5, CDFA’s recalibration algorithm successfully shifts the calibration curves closer to the diagonal line, thereby reducing the miscalibration area and improving the reliability of the predictions.

**FIGURE 6:**
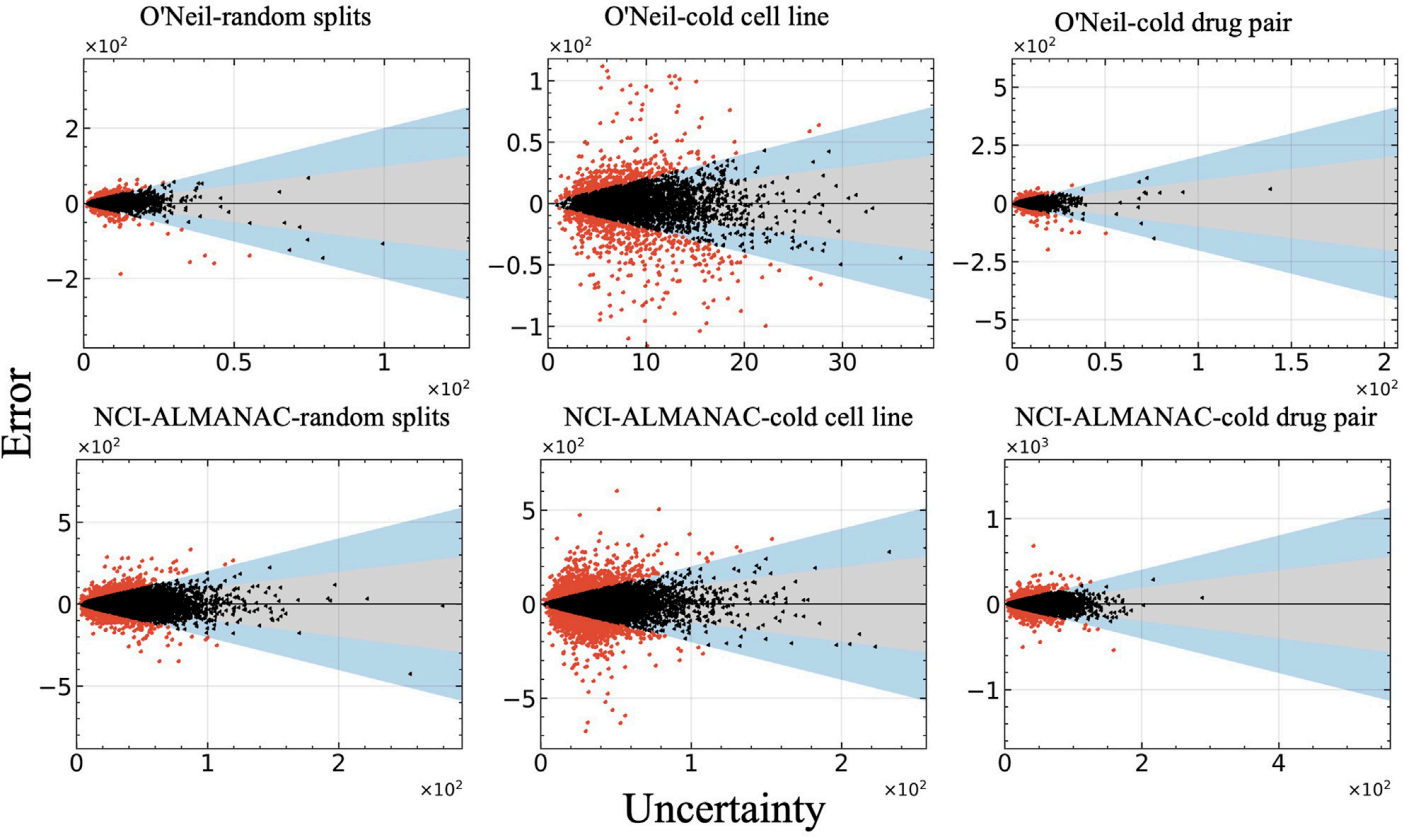
Relationship between model error and uncertainty of all test datasets. The vertical axis represents the prediction errors, while the horizontal axis displays uncertainty measured as the standard deviation (std).

Figure 6 illustrates the relationship between prediction error and uncertainty, with uncertainty measured as the standard deviation (std). In this figure, red points indicate errors that do not fall within two standard deviations, while black and blue points represent errors that fall within one and two standard deviations, respectively. It is evident that the majority of the observed errors lie within two standard deviations, reflecting a reasonable alignment between the model’s predicted uncertainty and its actual prediction error.

## 4 Conclusion

This study introduces an ensemble deep learning framework for predicting the potential synergy effects of drug combinations, showcasing superior performance relative to existing methods. A key innovation is the dual-level feature fusion mechanism, which integrates deep semantic features from various network modules, enhancing the model’s ability to capture complex interactions. The model leverages convolutional processing of the gene expression matrix to identify key gene signals relevant to drug response. Combined with a Transformer-based attention mechanism, this architecture enables context-aware re-weighting of gene importance under specific drug–cell interactions. This design emulates biological processes where only a subset of genes contribute significantly to the synergistic effect of drug combinations. Furthermore, the model’s prediction errors demonstrate robust generalization across tissues, as reflected in the consistent error distributions observed across different tissue types. Isolated high-error samples may correspond to biologically unique or complex cell lines, offering potential avenues for future investigation. Uncertainty estimation is integrated into the model, providing a critical safeguard against biased or overconfident predictions. This feature is especially valuable in guiding both the refinement of known synergies and the exploration of novel drug combinations. Additionally, the uncertainty estimation is integrated into the model, providing a critical safeguard against biased or overconfident predictions. This feature is especially valuable in guiding both the refinement of known synergies and the exploration of novel drug combinations. The uncertainty quantification and recalibration processes ensure that the model’s predictions are not only accurate but also reliable, offering a balanced approach to decision-making. While the experimental results demonstrate excellent performance on two datasets, further investigation is needed to assess the model’s robustness and generalization capabilities, particularly in scenarios involving new cell lines. Enhancing the interpretability of the model is another important area for future research, as it can provide deeper insights into the mechanisms underlying drug synergy and facilitate broader acceptance.

## Data Availability

The datasets presented in this study can be found in online repositories. The names of the repository/repositories and accession number(s) can be found in the article/supplementary material. All codes of CDFA can be accessed from https://github.com/TracyHIT/CDFA.
